# RETRACTION: A Novel Molecular Mechanism of IKKε‐Mediated Akt/mTOR Inhibition in the Cardiomyocyte Autophagy after Myocardial Infarction

**DOI:** 10.1155/omcl/9893408

**Published:** 2026-02-03

**Authors:** Oxidative Medicine and Cellular Longevity

RETRACTION: S. He, J. Shen, L. Li, Y. Xu, Y. Cao, L. Yin, Z. Tao, Z. Qiu, W. Chen, and X. Chen, “A Novel Molecular Mechanism of IKKε‐Mediated Akt/mTOR Inhibition in the Cardiomyocyte Autophagy after Myocardial Infarction,” *Oxidative Medicine and Cellular Longevity*, no. 2020 (2020). https://doi.org/10.1155/2020/7046923.

The above article, published online on 17 July 2020 in Wiley Online Library (wileyonlinelibrary.com), has been retracted following the agreement of the journal’s Chief Editor Jeannette Vasquez‐Vivar; and John Wiley & Sons Ltd.

The retraction has been agreed following an investigation of the concerns raised by *Actinopolyspora biskrensis* on PubPeer [1]. More specifically, unexpected similarities were identified between the first and third images of the H9c2 cells in the starvation group shown in Figure 5c. Additional similarity has been identified between the Western Blot bands representing the expression of LC3‐I/II in Sham and MI‐7d groups in Figure 1a and the p‐AKT expression in the starvation group shown in Figure 6e.

As a result of the investigation, the data of this article are considered unreliable.

The authors were informed of this retraction but did not provide a response.
